# Genetic Association Study Between Refractive Error–Related Genes and High Myopia in the Chinese Han Population

**DOI:** 10.1155/joph/8363695

**Published:** 2026-05-13

**Authors:** Jianxin Liu, Weijiang Liao, Chunbao Xie, Xinyu Wang, Yukai Gong, Chunchun Yang, Fang Hao, Yin Yang, Liang Zou, Yi Shi, Jiayu Meng, Lingxi Jiang

**Affiliations:** ^1^ Department of Laboratory Medicine, Sichuan Academy of Medical Sciences and Sichuan Provincial People’s Hospital, School of Medicine, University of Electronic Science and Technology of China, Chengdu, 610072, Sichuan, China, scu.edu.cn; ^2^ School of Food and Biological Engineering, Chengdu University, Chengdu, 610106, Sichuan, China, scu.edu.cn; ^3^ Department of Otolaryngology-Head and Neck Surgery, Sichuan Provincial People’s Hospital, School of Medicine, University of Electronic Science and Technology of China, Chengdu, 610072, Sichuan, China, scu.edu.cn; ^4^ Department of Ophthalmology, Sichuan Provincial People’s Hospital, School of Medicine, University of Electronic Science and Technology of China, Chengdu, 610072, Sichuan, China, scu.edu.cn; ^5^ Research Unit for Blindness Prevention of Chinese Academy of Medical Sciences (2019RU026), Sichuan Academy of Medical Sciences and Sichuan Provincial People’s Hospital, Chengdu, 610072, Sichuan, China, scu.edu.cn; ^6^ Sichuan-Chongqing Joint Key Laboratory of Pathology and Laboratory Medicine, Jinfeng Laboratory, Chongqing, 401329, China, scu.edu.cn

**Keywords:** C × 36, GJD2 gene, high myopia, rs580839

## Abstract

**Background:**

Refractive errors are the leading cause of visual impairment and blindness globally. High myopia (HM) poses significant risks of severe ocular complications and blindness. 12 SNPs, including rs580839, have been associated with refractive errors in European and Asian populations, but their roles in Chinese cohorts remain unexplored. Their genetic association with HM is investigated in this study.

**Methods:**

Genotyping of specific SNPs was conducted using multiplexed SNP/exome capture sequencing in a cohort consisting of HM patients and emmetropic individuals. The 3DSNP and GTEx databases were used to identify the causal association between SNPs and regulated genes. In addition, a mouse form–deprivation myopia (FDM) model was established, and retinal protein levels were quantified by the western blot analysis.

**Results:**

Our study revealed that the heterozygous genotype of *GJD2*‐rs580839 (G/A) was associated with HM in both the heterozygous (GA vs. GG: *p* = 0.021, OR = 1.433) and dominant (GA + AA vs. GG: *p* = 0.026, OR = 1.388) inheritance models. GTEx database indicates that the aforementioned risk genotypes (rs580839‐AG and rs580839‐AG + AA) are associated with the regulation of *GJD2* gene expression. In the FDM mouse model, a significant downregulation of C × 36 protein expression was observed.

**Conclusions:**

This study showed a statistically suggestive association between rs580839 (*GJD2*) and HM among the Han Chinese population. In addition, rs580839 may influence the pathogenesis of HM through the C × 36‐mediated pathway. However, further in vivo studies are required to elucidate the complex mechanisms that underlie HM development.

## 1. Introduction

Refractive errors (including astigmatism, hyperopia, and myopia) are eye conditions in which the optical system fails to focus light on the retina, which causes blurred vision [1]. Myopia is a subtype of refractive error that can be stratified into three categories (low, moderate, and high). Myopia currently represents the most pathogenic and prevalent refractive error and impacts the largest demographic. Previous studies have found that myopia affects approximately 75% of Southeast Asian adults, 30% of European adults, and 35% of American adults [2–4]. In addition to its high incidence, the rising trend and earlier onset age are crucial characteristics of myopia. In the last 30 years, the global epidemiological burden of myopia has exhibited a consistent upward trajectory, and there has been a notably elevated prevalence among adolescent populations; approximately one‐third of teenagers are affected by myopia [5]. The vast population of myopia patients presents a significant global challenge to vision health. Clinical studies have indicated that 10%–20% of myopic patients progress to high myopia (HM). It is notable that HM is considered to be an irreversible disease that is characterized by the increased axial length (AL) and severe refractive error (≤ −6.0 diopters [D]) [6, 7]. For these HM individuals, there is a significant risk for irreversible visual impairment, with common complications such as retinal detachment, macular retinoschisis, and choroidal neovascularization associated with pathological myopia [8, 9]. It is projected that the global prevalence of HM will substantially increase by 2050, with approximately 10% of the affected individuals progressing to HM. Therefore, HM is a critical global health priority.

The pathophysiology of myopia remains poorly understood despite its significant public health impact. Research efforts have increasingly focused on elucidating the etiology and pathogenesis of refractive errors and myopia as a means of enhancing preventive and therapeutic interventions. Current epidemiological and molecular studies have found a robust correlation between refractive anomalies, myopia, environmental exposures, and behavioral factors [10, 11]. However, familial aggregation patterns suggest a substantial genetic component in myopia susceptibility [12–14]. While most genetic association studies of refractive errors have been conducted in Caucasian populations, the prevalence and genetic architecture of myopia are notably more complex in Chinese populations [15]. It is noteworthy that a recent study has uncovered the genetic uniqueness of myopia in Chinese children. The research identified not only previously known genes such as TENM3 but also novel candidate loci including MIR4275 and FAM135B. Furthermore, it demonstrated a significant interaction between high genetic risk scores and extended periods of near‐work activities, substantially elevating the risk of developing myopia [16]. Therefore, the identification of novel genetic variants specific to Chinese populations and the characterization of the molecular genetic mechanisms driving myopia development are essential for advancing population‐specific prevention, management, and treatment strategies for refractive errors and myopia.

Two genomewide association studies (GWAS) by the Myopia Consortium and the Blue Mountains Eye Study (BMES) found 32 single‐nucleotide polymorphisms (SNPs) that are associated with refractive error among both Caucasian and Asian populations [17–19]. Among them, 20 SNPs have been validated within Han Chinese cohorts. For example, risk alleles of rs524952 (*GJD2*), rs7744813 (*KCNQ5*), and rs13382811 (*ZFHX1B*) have been implicated in the progression of myopia among children [20]. In addition, rs9307551 (*LOC100506035*), rs17648524 (*A2BP1*), rs7084402 (*BICC1*), rs634990 (*GJD2*), and rs8032019 (*GJD2*) have been previously linked to refractive errors among Han Chinese populations [21–24]. These 20 SNP reports have contributed to the advancement of genetic association studies on myopia‐related loci among the Chinese population and have significant implications for the development of preventive and therapeutic strategies for myopia. The remaining 12 SNPs, which include rs14165 (*CACNA1D*), rs4237036 (*CHD7*), rs10882165 (*CYP26A1*), rs2184971 (*PCCA*), rs8000973 (*ZIC2*), rs17183295 (*MYO1D*), rs4793501 (*KCNJ2*), rs12971120 (*CNDP2*), rs11145465 (*TJP2*), rs235770 (*BMP2*), rs560766 (*GJD2*), and rs580839 (*GJD2*), remain unvalidated in Chinese populations. This study focused on the remaining 12 SNP loci for validation in a Han Chinese sample that consisted of 508 HM cases and 500 healthy controls.

## 2. Methods

### 2.1. Research Population

508 HM patients and 500 healthy controls were recruited from Sichuan Provincial People’s Hospital. The experimental protocols involving human subjects and animal models in this study have received approval from the Institutional Review Board of Sichuan Academy of Medical Sciences and Sichuan Provincial People’s Hospital and are conducted in strict accordance with the ethical principles outlined in the Declaration of Helsinki (ethical committee number: 2024‐287). All participants provided written informed consent before initiation. All subjects underwent comprehensive ophthalmic assessments. The AL of all highly myopic eyes was ≥ 26.0 mm. All participants had no history of lens, corneal pathology, or refractive surgery. Participants in the control group had no refractive errors or ocular pathologies in either eye. All samples and associated data were anonymized to ensure participant confidentiality.

### 2.2. SNP Selection

As previously mentioned, two GWAS studies have identified 32 SNPs associated with refractive error phenotypes among Caucasian and Asian populations. In one GWAS, 28 SNPs were found to be related to refractive errors and myopia. In addition, two other studies pinpointed four SNPs within the 15q14 region that are associated with refractive error susceptibility among the Caucasian population (rs560766, rs634990, rs580839, and rs8032019) [17–19]. These three studies have provided guidance for subsequent genetic association studies.

Of the 32 SNPs, the association of 20 loci with refractive errors/myopia among Han Chinese populations has been independently verified. This study chose the remaining 12 additional untested SNPs, rs560766 (*GJD2*), rs580839 (*GJD2*), rs14165 (*CACNA1D*), rs4237036 (*CHD7*), rs10882165 (*CYP26A1*), rs2184971 (*PCCA*), rs8000973 (*ZIC2*), rs17183295 (*MYO1D*), rs4793501 (*KCNJ2*), rs12971120 (*CNDP2*), rs11145465 (*TJP2*), and rs235770 (*BMP2*), to investigate their potential association with HM among a Han Chinese cohort.

### 2.3. Genotype Analysis

Venous blood samples were collected from all participants and placed in anticoagulant tubes. Genomic DNA was extracted using a blood genomic DNA extraction kit (Sangon Biotech, Shanghai, China). A panel was designed for specific SNP loci, and the target SNP sequences were amplified using a two‐step PCR method. Sequencing libraries were then prepared, quantified, pooled in equal amounts, and sequenced on the Illumina HiSeq X Ten platform. Raw sequencing data were processed to remove adapter sequences and perform quality filtering. The resulting high‐quality reads were aligned to the reference genome using the BWA software. Genotyping at the target loci was performed using SAMtools, and mutation annotation was conducted with ANNOVAR software [25–27].

### 2.4. 3DSNP and GTEx Analysis

The three‐dimensional chromatin interaction database (3DSNP) (https://omic.tech/3dsnpv2/) is a comprehensive resource that systematically describes the way in which human noncoding SNPs regulate gene expression. This is achieved by investigating chromatin loop–mediated 3D contacts between these SNPs and their target genes, in addition to other functionally related SNPs [28]. The Genotype‐Tissue Expression (GTEx) (https://www.gtexportal.org/home/) database helps interpret GWAS results that are used in translational research by providing extensive eQTL data across numerous tissues associated with a variety of diseases [29]. In addition, GTEx analysis is able to identify the regulatory effect of SNP loci on gene expression, which lies in its systematic establishment of causal associations between genetic variation SNPs and gene expression by integrating large‐scale multiomics data and statistical genetics methods. This study utilized both 3DSNP and GTEx to score each SNP to find the causal associations between SNPs and regulated genes.

### 2.5. Form‐Deprivation Myopia (FDM) Model Construction

All experimental mice were housed in SPF‐grade barrier environments, and this study protocol received approval from the Medical Ethics Committee of Sichuan Academy of Medical Sciences and Sichuan Provincial People’s Hospital (ethical committee number: 2024‐287). The neck size of three‐week‐old inbred SPF‐grade healthy male C57BL/6 mice (purchased from Chengdu GemPharmatech Co. Ltd) was measured. A 3‐month adhesive tape was used to cut a collar to prevent the mice from scratching their eyes. Based on the periocular size, a semitransparent plastic sheet was fabricated into an eye mask with an approximate diameter of 8 mm and an approximate peripheral edge of 1 mm for sensory deprivation. The ocular condition of the mice was examined three times a day in the morning, at noon, and in the evening to check for any abnormal secretions, infections, or dislodgement of the eye mask. It was ensured that the mice remained in a state of sensory deprivation throughout the entire experiment [30, 31].

### 2.6. Western Blotting

Standard immunoblotting methods were employed. Retinal proteins were extracted with high‐efficiency radio immunoprecipitation assay (RIPA) buffer and sonication, and protein concentration was determined using an enhanced Bicinchoninic Acid (BCA) Protein Assay Kit (Beyotime, Shanghai, China). Equal amounts of protein (10 μg) were separated by 10% sodium dodecyl sulfate–polyacrylamide gel electrophoresis (SDS‐PAGE). The separated proteins were then transferred onto nitrocellulose membranes (Epizyme, China). The membranes were blocked with 5% skim milk for 2 h and incubated with primary antibodies overnight at 4°C [32]. The primary antibodies included Connexin 36 (C × 36; 1:2000, Bioss, bs‐1266R) and β‐actin (1:5000, Proteintech, 60008‐1‐Ig) as internal controls. The membranes were washed in Tris‐buffered saline with Tween 20 (TBST) (Servicebio, Wuhan, China) and then incubated with HRP‐conjugated goat anti‐rabbit IgG (1:5000, Cell Signaling, 7074S) or HRP‐conjugated goat anti‐mouse IgG secondary antibody (1:5000, Cell Signaling, 7076S) for 1 h at room temperature. Visualization was performed using an enhanced chemiluminescence (ECL) detection kit (Biosharp, China), and band densities were quantified using ImageJ.

### 2.7. Statistical Analysis

The association between SNP and HM was analyzed across allelic, genotypic, and other inheritance models (homozygous, heterozygous, dominant, and recessive) using chi‐square tests and Bonferroni multiple‐correction tests (*p* value < 0.05 were deemed statistically significant) [33]. For SNP loci exhibiting significant associations, binary logistic regression analysis was performed using SPSS 26.0, with age and sex incorporated as covariates for adjustment. Genotype and allele frequency tables for cases and controls were constructed for genetic association analysis. Minor allele frequency (MAF), odds ratio (OR) for the minor allele, and 95% confidence intervals (95% CIs) were calculated as a means of estimating the effect size of the minor allele on HM.

To analyze the data from animal experiments, this study employed GraphPad Prism 10.0 statistical software, with results expressed as mean ± SD. Comparative analyses between groups were performed using Student′s *t*‐test, with a significance threshold set at *p* < 0.05.

## 3. Results

1008 unrelated participants were recruited for this study: 508 individuals with HM and 500 controls. The average spherical refractive error in patients with HM was −10.683 ± 6.062 D in the right eye (OD) and −10.066 ± 5.528 D in the left eye (OS). The AL value in patients with HM was 28.851 ± 2.41 mm (range: 22.12–35.90 mm) and 28.737 ± 2.381 mm (range: 22.19–34.97 mm). Patient age ranged from 3 to 89 years (30.75 ± 21.13), and male patients accounted for 55.35% of the patient population. Control subject age ranged from 18 to 87 years (45.55 ± 13.11), and male subjects accounted for 53.84% of the controls. Other demographic data of all subjects can be seen in Table 1.

**TABLE 1:** Clinical characteristics of HM patients and the control group in the study population.

Group	Number	Average age (years old)	Gender		Axial length (mm)		Refractive error (D)	
			Male (number)	Female (number)	OD	OS	OD	OS
Patients	508	30.75 ± 21.13	262	246	28.851 ± 2.410	28.737 ± 2.381	−10.683 ± 6.062	−10.066 ± 5.528
Control	500	45.55 ± 13.11	248	252	≤ 26.0	≤ 26.0	< ± 0.5	< ± 0.5

In contrast to the previous two GWAS studies, neither the case group nor the control group exhibited detection of the three SNPs (rs11145465, rs10882165, and rs17183295). Therefore, the genotyping was performed on nine additional SNPs, followed by Hardy–Weinberg equilibrium (HWE) analysis. Four SNPs (rs14165, rs4237036, rs2184971, and rs12971120) deviated from HWE (*p* < 0.001) by displaying different genotypes than anticipated. Preliminary results found rs580839 and rs560766 to have a significant association with HM (rs580839: *p* = 0.017, OR = 1.239, 95% CI = 1.039‐1.478; rs560766: *p* = 0.049, OR = 1.193, 95% CI = 1.001–1.423) among the remaining five SNPs. The MAF of rs580839‐A was 0.409 in control subjects and 0.462 in individuals with HM. For rs560766‐A, the MAF was 0.420 in control subjects and 0.464 in individuals with HM (Table 2). These MAF values were consistent with the NCBI data (Supporting Tables 1 and 2).

**TABLE 2:** Association of nine SNPs in the Han ethnic group with HM.

SNP	Chromosome	Position	Gene	Minor allele	MAF		*p*‐HWE		Allelic *p* ^a^	OR (95% CI)^b^
					Control	Case	Control	Case		
rs14165	3	53847408	*CACNA1D*	A	0.716	0.138	0.000	0.000	0.000	0.063 (0.051–0.079)
rs4237036	8	61701057	*CHD7*	T	0.500	0.489	0.000	0.000	0.627	0.958 (0.804–1.140)
rs8000973	13	100691367	*ZIC2*	C	0.234	0.248	0.877	0.952	0.461	1.080 (0.880–1.324)
rs2184971	13	100818092	*PCCA*	A	0.695	0.307	0.000	0.000	0.000	0.194 (0.161–0.235)
**rs580839**	15	34998829	*GJD2*	A	0.409	0.462	0.420	0.265	**0.017**	1.239 (1.039–1.478)
**rs560766**	15	35000942	*GJD2*	A	0.420	0.464	0.378	0.360	**0.049**	1.193 (1.001–1.423)
rs4793501	17	68718734	*KCNJ2*	T	0.351	0.359	0.610	0.621	0.699	1.037 (0.864–1.244)
rs12971120	18	72174023	*CNDP2*	G	0.238	0.243	0.005	0.000	0.788	1.028 (0.838–1.261)
rs235770	20	6761765	*BMP2*	C	0.277	0.314	0.598	0.145	0.069	1.194 (0.986–1.447)

*Note:* Bold text indicates SNPs with statistical significance (*p* < 0.05).

^a^Allelic *p* value was not adjusted by age and sex.

^b^ORs (95% CI) were adjusted by age and sex and were determined by the *χ* ^2^ test, patients versus controls.

Five genetic models (allelic, homozygous, heterozygous, dominant, and recessive models) were applied for further evaluation of the potential correlation between the SNPs and HM. After adjustments for age and sex via SPSS, the heterozygous and dominant models of rs580839 demonstrated statistical significance (*p* < 0.05), while none of the five rs560766 models showed significant association after correction. The results without Bonferroni correction indicate that individuals who were carrying rs580839‐GG had a lower risk of HM compared to carriers of rs580839AA and rs580839 AA + AG (*p* = 0.021, OR = 1.433, 95% CI = 1.056–1.942; *p* = 0.026, OR = 1.388, 95% CI = 1.040–1.861). However, after Bonferroni correction for multiple testing, the correlations did not reach statistical significance within the corresponding genetic models (respectively, Table 3). Genotype distribution analysis (Table 4) showed that the frequencies of the rs580839‐AA (0.201) and rs580839‐AG (0.522) genotypes in the HM patient group were slightly higher than those in control subjects (AA: 0.176; AG: 0.466), while the GG genotype (0.278) was lower than that in control subjects (0.358).

**TABLE 3:** SPSS correction results for five genetic models of rs580839 and rs560766.

SNP	Model	Adjusted OR (95% CI)^a^	Adjusted *p* ^b^	Adjusted *p* ^c^
rs580839	Allelic	1.164 (0.961–1.409)	0.121	0.605
	Homozygous	1.266 (0.857–1.870)	0.236	1
	Heterozygous	1.433 (1.056–1.942)	**0.021**	0.105
	Dominant	1.388 (1.040–1.851)	**0.026**	0.130
	Recessive	1.020 (0.724–1.437)	0.909	1

rs560766	Allelic	1.121 (0.926–1.357)	0.240	1
	Homozygous	1.191 (0.809–1.753)	0.376	1
	Heterozygous	1.348 (0.992–1.831)	0.056	0.280
	Dominant	1.304 (0.976–1.741)	0.072	0.360
	Recessive	0.995 (0.710–1.394)	0.977	1

*Note:* Bold text indicates the SNPs with statistical significance (*p* < 0.05).

^a^Adjusted OR (95% CI) value was obtained by adjusting for age and sex.

^b^Adjusted *p* value was obtained by adjusting for age and sex.

^c^Bonferroni multiple‐correction tests: Adjusted *p* = *p* × 5 (the number of models).

**TABLE 4:** Genotype distribution of rs580839.

Group	Genotype (*n*%)			Total (n)
	AA	AG	GG	
Control	88 (0.176)	233 (0.466)	179 (0.358)	500
Patients	102 (0.201)	265 (0.522)	141 (0.277)	508

An exploratory analysis was then performed to investigate the potential rs580839‐regulated genes. 3DSNP data indicated that rs580839 strongly interacts with the *GJD2* gene in the same chromatin regulatory loops (Supporting Table 3 and Supporting Figure 1). In addition, GTEx analysis systematically revealed the regulatory mechanism of SNPs on gene expression through large‐scale multiomics data integration, statistical genetics methods, and functional experimental verification. In the GTEx database, it was found that people who carry the rs580839‐AA/AG (HM risk genotypes) have lower *GJD2* gene expression levels than those who carry the rs580839‐GG genotype (HM protective genotype) in up to four human tissues (Supporting Table 4 and Figure 1).

FIGURE 1Expression levels of *GJD2* and its transcript *GJD2-DT* across three genotypes, based on tissue‐specific eQTL analysis of the rs580839 locus (GTEx database). (a) GJD2 in the pituitary. (b) GJD2 in the pancreas. (c) GJD2 in the brain–cerebellum. (d) GJD2‐DT in the pituitary. (e) GJD2‐DT in the artery–tibia.

**Figure:**
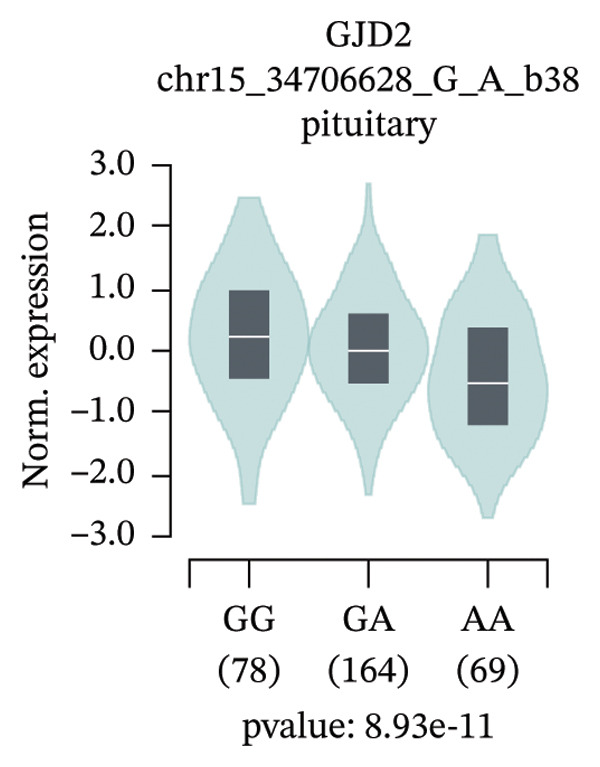
(a)

**Figure:**
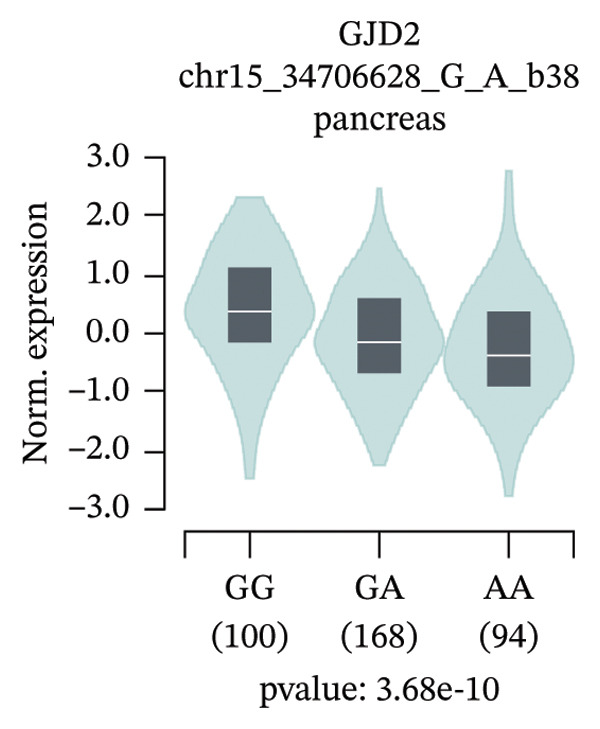
(b)

**Figure:**
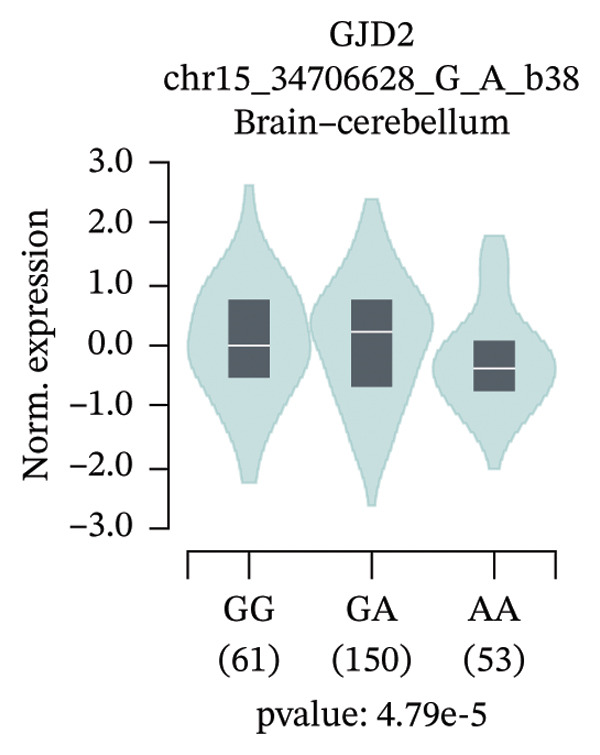
(c)

**Figure:**
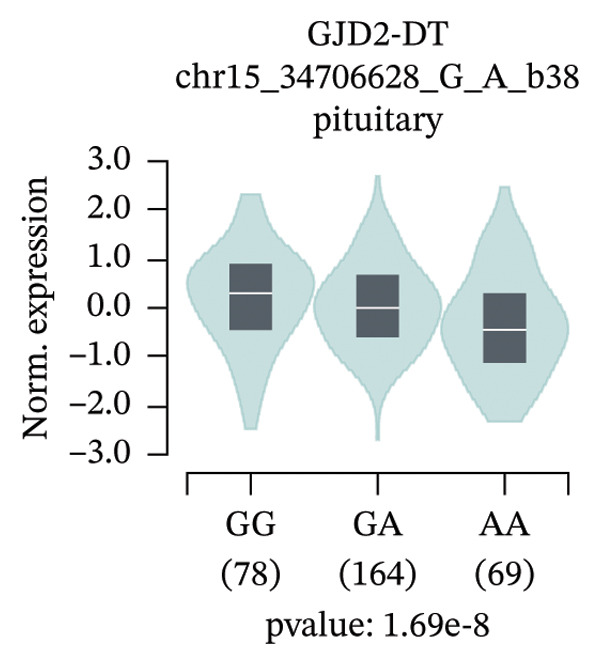
(d)

**Figure:**
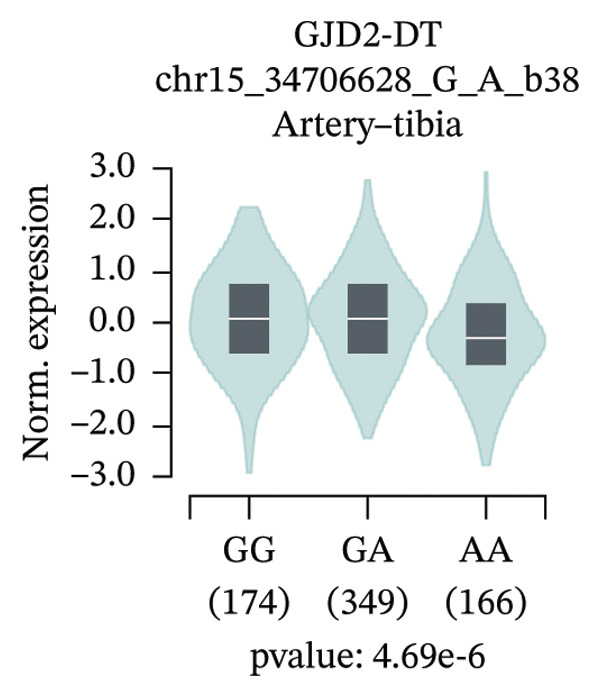
(e)

Generally, both the 3DSNP and GTEx human databases revealed that rs580839 has a high likelihood of directly regulating *GJD2* expression. HM risk genotype rs580839‐AA and rs580839‐AG carriers could have a lower expression of *GJD2*. A mouse myopia model (FDM) was constructed to investigate whether decreased Cx36 (encoded by *GJD2*) can be regarded as an important marker of myopia. Interestingly, a significant downregulation of C × 36 was observed in the retinal tissues of the FDM model (Figure 2).

FIGURE 2Western blotting results of C × 36 expression levels in the native and FDM groups (*n* = 3).

**Figure:**
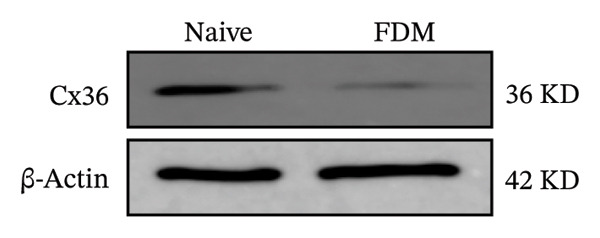
(a)

**Figure:**
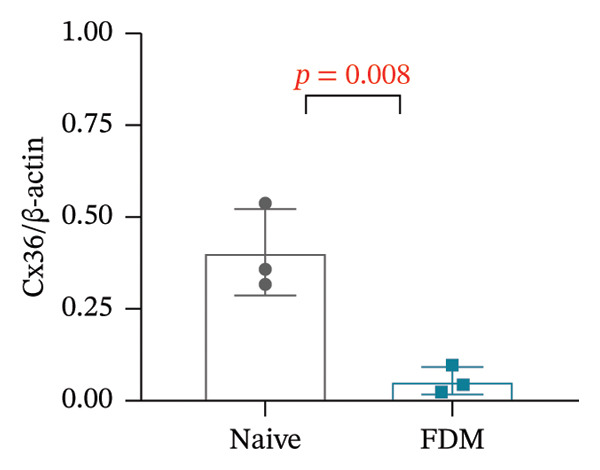
(b)

## 4. Discussion

Previous studies found that 12 SNPs may be risk factors for myopia among Caucasian and certain Asian populations [17–19]. However, these had not been verified among the Han Chinese population, who have an incredibly high HM incidence rate. This study conducted genotyping of 12 SNPs within a Han Chinese population from Western China and assessed their association with myopia. The results indicate that 11 SNP loci within this cohort show no significant association with myopia, with only the rs580839 locus demonstrating a significant correlation. It is notable that previous studies have reported significant associations between the SNPs rs11145465, rs10882165, and rs17183295 and HM in European populations; however, these SNPs have not detected the same trend in this study cohort [17]. This highlights the genetic evolutionary differences that exist among populations and suggests that substantial population‐specific genetic factors influence HM [15]. As a consequence, conducting population‐specific genetic association analyses remains both essential and meaningful.

This study reveals a significant association between rs580839 and HM in Han Chinese populations, prior to correction for multiple testing. However, following Bonferroni correction for multiple comparisons, this association did not reach the threshold of statistical significance. In addition, the functional annotation via 3DSNP and GTEx databases revealed that it is highly likely to regulate the expression of *GJD2* directly. Further analysis of GTEx data revealed that individuals carrying the rs580839 minor allele (A) exhibit a significant reduction in *GJD2* (encoding Connexin 36, Cx36) expression levels. This finding suggests that decreased *GJD2* expression may serve as a critical etiological factor in the development of HM. Mechanistically, this underpins the necessity to examine Cx36 expression alterations in animal models. Thus, we provide the first evidence suggesting a potential link between rs580839 and myopia susceptibility within the Han Chinese population, with rs580839‐A carriers exhibiting concomitant downregulation of Cx36 protein expression.

The *GJD2* gene encodes the Cx36 protein, and it has garnered significant attention due to the role it plays in myopia pathogenesis. Several independent studies have validated *GJD2* as an early genetic locus that is associated with myopia in diverse cohorts and ethnicities [17, 18, 34, 35]. Cx36 is extensively expressed in the retina, including cone photoreceptors, all amacrine cells, bipolar cells, and other photoreceptors [36]. The dysfunction of Cx36 is believed to disrupt the balance of ON and OFF visual pathways, which could potentially affect refractive development [37]. A study indicated that the *GJD2* risk genotype primarily contributes to myopia via a dose‐dependent mechanism, and this results in increased vitreous chamber depth (VCD) and anterior chamber depth (ACD) during early developmental stages [38]. Knockout models of *GJD2* orthologs in zebrafish resulted in refractive errors [39], and significant reductions in *GJD2* mRNA and Cx36 protein levels were observed in guinea pig FDM models [40]. Meanwhile, this study further validated in the C57BL/6 murine myopia model that Cx36 expression remains diminished. Consequently, the downregulation of Cx36 is confirmed as a significant factor in the pathogenesis and progression of myopia across three distinct experimental models (Figure 2).

Our study demonstrated that under heterozygous and dominant inheritance models, the rs580839 locus exhibited statistical significance prior to multiple testing correction. However, this significance was lost following Bonferroni correction, indicating that neglecting such adjustments may inflate the risk of Type I errors. Nevertheless, integrating the following biological evidence suggests that this association warrants further investigation: first, analysis of the GTEx database indicates that rs580839 may directly regulate *GJD2* expression; second, the *GJD2*‐encoded protein, Cx36, has been consistently shown to be downregulated in various myopia animal models [39, 40]. Due to the statistical limitations, our study added the convergence of genomic and functional evidence to provide complementary support for the potential role of this locus.

This study has limitations. First, demographic differences (age and sex) existed between cases and controls. We addressed this potential confounding by including these variables as covariates in our multivariate models, although residual confounding cannot be entirely excluded. Future studies would benefit from more rigorously matched cohorts. Second, deviations from the HWE were observed for some SNPs in controls. While genotyping errors were unlikely, the modest sample size is a plausible explanation. Although our primary analysis accounted for genotype distributions, the HWE deviation warrants caution. Our findings require validation in larger, independent cohorts.

In summary, this study has found a significant association between *GJD2-*rs580839 and HM in the Han Chinese population prior to correction for multiple testing and found that the lower *GJD2* (Cx36) expression could be a strong risk factor for HM. The integration of 3DSNP, GTEx, and Western blot analyses hints that the SNP rs580839 might be involved in HM progression by downregulating Cx36 expression. However, it is important to note that after rigorous multiple testing correction, the genetic association did not retain formal statistical significance. Therefore, the current evidence primarily establishes preliminary correlative links. In addition, direct functional validation through gene editing or other molecular interventions is still needed to clarify the specific role of Cx

## Author Contributions

Yi Shi and Lingxi Jiang contributed significantly to the study’s conceptualization and experimental design. Jianxin Liu, Weijiang Liao, and Xinyu Wang were responsible for the acquisition of clinical data from HM patients. Liang Zou, Chunchun Yang, Yukai Gong, and Jiayu Meng provided technical support and guidance on analytical software. Yin Yang, Fang Hao, and Chunbao Xie conducted comprehensive ophthalmologic evaluations. Jianxin Liu, Weijiang Liao, and Lingxi Jiang performed data analysis, interpretation, and played a leading role in manuscript preparation.

## Funding

This work was supported by the National Natural Science Foundation of China (Grant nos. 82571260 to Lingxi Jiang, 82271120 and 82121003 to Yi Shi, and 82401244 to Jiayu Meng), the Sichuan Science and Technology Program (Grant no. 2025YFHZ0240 to Lingxi Jiang), the TOP Young Talents Program of Sichuan (Grant no. DQ202407 to Yi Shi), and the Chengdu Science and Technology program (Grant no. 2025‐YF09‐00047‐SN).

## Disclosure

All authors reviewed and approved the final version of the manuscript.

## Ethics Statement

The experimental protocols involving human subjects and animal models in this study have received approval from the Institutional Review Board of Sichuan Academy of Medical Sciences and Sichuan Provincial People’s Hospital and are conducted in strict accordance with the ethical principles outlined in the Declaration of Helsinki (ethical committee number: 2024‐287). Written informed consent was obtained from all participants prior to enrollment.

## Consent

Please see the Ethics Statement.

## Conflicts of Interest

The authors declare no conflicts of interest.

## Supporting Information

Additional supporting information can be found online in the Supporting Information section.

## Supporting information


**Supporting Information** Supporting Table 1: Global allele frequency distribution of rs580839‐A in diverse populations from NCBI databases. Supporting Table 2: Global allele frequency distribution of rs560766‐A in diverse populations from NCBI databases. Supporting Table 3: The interaction gene of rs580839 in the 3DSNP database. Supporting Table 4: GTEx‐based functional annotation of rs580839 eQTL effects. Supporting Figure 1: Visualization of rs580839 in the three‐dimensional chromatin interaction database.

## Data Availability

All data generated or analyzed during this study are included in this published article.
