# Supplementary material for: Genetic Association Study Between Refractive Error–Related Genes and High Myopia in the Chinese Han Population
Source: J Ophthalmol. 2026 May 13;2026:8363695. doi: 10.1155/joph/8363695 (PMC13172293; doi:10.1155/joph/8363695)
Supplement: Supplementary file 1 — Supporting Information Supporting Table 1: Global allele frequency distribution of rs580839‐A in diverse populations from NCBI databases. Supporting Table 2: Global allele frequency distribution of rs560766‐A in diverse populations from NCBI databases. Supporting Table 3: The interaction gene of rs580839 in the 3DSNP database. Supporting Table 4: GTEx‐based functional annotation of rs580839 eQTL effects. Supporting Figure 1: Visualization of rs580839 in the three‐dimensional chromatin interaction database. [file JOPH-2026-8363695-s001.zip › Supplementary Table/Supplementary Table 1.pdf]

Supplementary Table 1 Global allele frequency distribution of rs580839-A in diverse populations from NCBI databases.

Population	Group	Alt Allele
Total	Global	A=0.46132
European	Sub	A=0.42164
African	Sub	A=0.6877
African Others	Sub	A=0.679
African American	Sub	A=0.6880
Asian	Sub	A=0.433
East Asian	Sub	A=0.413
Other Asian	Sub	A=0.47
Latin American 1	Sub	A=0.467
Latin American 2	Sub	A=0.3785
South Asian	Sub	A=0.509
Other	Sub	A=0.4281
