# Supplementary material for: Genetic Association Study Between Refractive Error–Related Genes and High Myopia in the Chinese Han Population
Source: J Ophthalmol. 2026 May 13;2026:8363695. doi: 10.1155/joph/8363695 (PMC13172293; doi:10.1155/joph/8363695)
Supplement: Supplementary file 1 — Supporting Information Supporting Table 1: Global allele frequency distribution of rs580839‐A in diverse populations from NCBI databases. Supporting Table 2: Global allele frequency distribution of rs560766‐A in diverse populations from NCBI databases. Supporting Table 3: The interaction gene of rs580839 in the 3DSNP database. Supporting Table 4: GTEx‐based functional annotation of rs580839 eQTL effects. Supporting Figure 1: Visualization of rs580839 in the three‐dimensional chromatin interaction database. [file JOPH-2026-8363695-s001.zip › Supplementary Table/Supplementary Table 2.pdf]

Supplementary Table 2 Global allele frequency distribution of rs560766-A in diverse populations from NCBI databases.

Population	Group	Alt Allele
Total	Global	A=0.44359
European	Sub	A=0.42949
African	Sub	A=0.5446
African Others	Sub	A=0.547
African American	Sub	A=0.5445
Asian	Sub	A=0.420
East Asian	Sub	A=0.403
Other Asian	Sub	A=0.46
Latin American 1	Sub	A=0.451
Latin American 2	Sub	A=0.3836
South Asian	Sub	A=0.5844
Other	Sub	A=0.4245
