# Supplementary material for: Genetic Association Study Between Refractive Error–Related Genes and High Myopia in the Chinese Han Population
Source: J Ophthalmol. 2026 May 13;2026:8363695. doi: 10.1155/joph/8363695 (PMC13172293; doi:10.1155/joph/8363695)
Supplement: Supplementary file 1 — Supporting Information Supporting Table 1: Global allele frequency distribution of rs580839‐A in diverse populations from NCBI databases. Supporting Table 2: Global allele frequency distribution of rs560766‐A in diverse populations from NCBI databases. Supporting Table 3: The interaction gene of rs580839 in the 3DSNP database. Supporting Table 4: GTEx‐based functional annotation of rs580839 eQTL effects. Supporting Figure 1: Visualization of rs580839 in the three‐dimensional chromatin interaction database. [file JOPH-2026-8363695-s001.zip › Supplementary Table/Supplementary Table 3.pdf]

Supplementary Table 3: The interaction gene of rs580839 in 3DSNP database.

Gene	Loop type	Loop start	Loop end	Distance	Cell types
GJD2	Within loop	chr15:34982201-34992201	chr15:35182201-35192201	200000	RPMI7951
GJD2	Within loop	chr15:34972201-34982201	chr15:35112201-35122201	140000	Cortex_DLPFC
GJD2	Within loop	chr15:34962201-34972201	chr15:35162201-35172201	200000	G401
GJD2	Within loop	chr15:34952201-34962201	chr15:35042201-35052201	90000	VentricleLeft
GJD2	Within loop	chr15:34952201-34962201	chr15:35112201-35122201	160000	PANC1
GJD2	Within loop	chr15:34992201-35002201	chr15:35182201-35192201	190000	A549
GJD2	Within loop	chr15:34992201-35002201	chr15:35142201-35152201	150000	HAP1_DKO
GJD2	Within loop	chr15:34982201-34992201	chr15:35132201-35142201	150000	Liver
GJD2	Within loop	chr15:34932201-34942201	chr15:35062201-35072201	130000	Caki2
