# Supplementary material for: Genetic Association Study Between Refractive Error–Related Genes and High Myopia in the Chinese Han Population
Source: J Ophthalmol. 2026 May 13;2026:8363695. doi: 10.1155/joph/8363695 (PMC13172293; doi:10.1155/joph/8363695)
Supplement: Supplementary file 1 — Supporting Information Supporting Table 1: Global allele frequency distribution of rs580839‐A in diverse populations from NCBI databases. Supporting Table 2: Global allele frequency distribution of rs560766‐A in diverse populations from NCBI databases. Supporting Table 3: The interaction gene of rs580839 in the 3DSNP database. Supporting Table 4: GTEx‐based functional annotation of rs580839 eQTL effects. Supporting Figure 1: Visualization of rs580839 in the three‐dimensional chromatin interaction database. [file JOPH-2026-8363695-s001.zip › Supplementary Table/Supplementary Table 4.pdf]

Supplementary Table 4: GTEx-based functional annotation of rs580839 eQTL effects.

Gene	*p*-value	NES [b]	Tissue
GJD2	8.93e-11	-0.34	Pituitary
GJD2	3.68e-10	-0.21	Pancreas
GJD2	4.79e-5	-0.27	Brain - Cerebellum
GJD2-DT[c]	1.69e-8	-0.27	Pituitary
GJD2-DT	4.69e-6	-0.19	Artery - Tibial

[a]***p***-value: Beta distribution-adjusted empirical p-values from FastQTL were used for calculating q-values (Storey & Tibshirani, PNAS, 2003), and a false discovery rate (FDR) threshold of ≤0.05 was applied for identifying genes with a significant eQTL.

[b]The NES (Normalized Effect Size) is defined as the slope of the linear regression and is computed as the effect of the alternative allele (ALT) relative to the reference allele (REF) in the human genome reference GRCh38/hg38.

[c]*GJD2-DT*:the divergent transcript of *GJD2*.
